# Quality Indicators of Pharmaceutical Care in Palestinian Integrative Healthcare Facilities: Findings of a Qualitative Study among Stakeholders

**DOI:** 10.1155/2020/4520769

**Published:** 2020-05-10

**Authors:** Ramzi Shawahna

**Affiliations:** ^1^Department of Physiology, Pharmacology and Toxicology, Faculty of Medicine and Health Sciences, An-Najah National University, Nablus, State of Palestine; ^2^An-Najah BioSciences Unit, Centre for Poisons Control, Chemical and Biological Analyses, An-Najah National University, Nablus, State of Palestine

## Abstract

**Background:**

Recently, there has been shifts from providing large volumes to providing higher quality of healthcare services. This qualitative exploratory study was conducted to explore the views of different stakeholders on activities and services that could serve as quality indicators of pharmaceutical care in Palestinian integrative healthcare facilities.

**Methods:**

A judgmental sampling technique was used to invite and recruit stakeholders for this study. Semistructured in-depth interviews were conducted with the stakeholders. Data collected during the interviews were qualitatively analyzed using the interpretive description methodology. Themes, subthemes, and patterns were recognized using the Qualitative Analysis Guide of Leuven. The data were coded using RQDA software.

**Results:**

Interviews (*n* = 22) were conducted with 9 complementary and alternative medicine practitioners, 8 pharmacists, 2 physicians, 2 nurses, and 1 risk/quality assurance manager. The interview median duration was 41 with an IQR of 22 min. Following the thematic analysis adopted to achieve the objectives of this study, six major themes emerged from the data collected from the interviews. The themes emerged from the data were (1) provision of collaborative, direct, and comprehensive patient care services; (2) common services and activities at the time of admission, during stay, at transition between wards/services/hospitals, and at discharge to home or community care; (3) screening for, identifying, and resolving problems; (4) collaboration with other healthcare providers; (5) professional development; and (6) performance and efficiency.

**Conclusions:**

Quality indicators are invaluable for informing decisions relevant to justifying allocation of scarce resources, securing funds, and demonstrating value in activities and services within integrative healthcare facilities. Further studies are still needed to develop a set of measurable indicators to measure the impact of pharmaceutical care in integrative healthcare facilities.

## 1. Introduction

Integrative medicine has emerged as a holistic approach to healthcare in which different forms of complementary and alternative medicine (CAM) are integrated in conventional Western medicine [1, 2]. Blending CAM within Western medicine has allowed healthcare providers to develop individualized care plans, catering to the different needs of patients, and provide holistic healthcare services that incorporate the mind, body, spirituality, and a sense of belonging to the community [1–3]. Recently, the concept of integrative medicine has gained considerable acceptance in societies embracing cultural diversity.

Recent studies have shown that catering to the different needs of patients through integrative medicine paradigms reduced morbidity, mortality, and costs of healthcare as well as improved quality of life of patients who suffered different diseases such as stroke [4], cardiovascular [5], cancer [6], inflammatory [7], and other diseases [8, 9].

In modern healthcare delivery systems, services are provided by different providers such as physicians, pharmacists, nurses, and CAM practitioners. Recently, competencies of pharmacists in providing healthcare services in integrative medicine have been recognized [10]. In contemporary healthcare systems, pharmacists are playing ever expanding roles and responsibilities that were advocated by many professional associations, notably those in the United States, United Kingdom, Australia, and Canada [11–15].

Different modalities of CAM are increasingly used by patients either to complement or as alternatives to their conventional Western medicines [15, 16]. In today's healthcare systems, the pharmacist is one of the main providers of CAM. Additionally, pharmacists are supposed to design therapeutic plans, order laboratory tests, screen for, identify, explain, and resolve drug-related problems including adverse drug reactions and drug interactions. Pharmacists are also supposed to monitor outcomes of the therapeutic plans, rectify ineffective therapies, and educate patients on how to make the best out of their therapeutic modalities [15–18].

Recently, there has been shifts from providing large volumes to providing higher quality of healthcare services [19–22]. Healthcare facilities employing integrative medicine are no exception and measuring performance and provision of healthcare services have drawn considerable attention. Quality measures to be used in assessing performance of healthcare providers and delivery of services across the continuum of healthcare were developed [19, 23]. In general, these quality measures might help ensure that services were provided consistently and efficiently [19, 20, 22].

Quality measures are often used by decision makers to justify allocating scarce resources, improve quality of services, bridge gaps, attract funds, improve accountability, and patient safety [20]. In a recent study, a core set of consensual key performance indicators that can serve in capturing the impact of pharmacists while providing care for patients with epilepsy in primary healthcare settings was developed [19]. Another core list of consensual key performance indicators that can serve in capturing performance of clinical pharmacists while caring for patients in hospitalized patient settings was developed [22]. A recent scoping review identified 42 different pharmaceutical activities and services that can be used as to measure quality of pharmaceutical care for integrative healthcare [24]. The activities and services that were developed using the Delphi technique included taking medication history, medication reconciliations, resolving therapy-related problems, provision of collaborative care, formulating therapeutic plans, optimal performance, and continuing education.

Little was narrated on the quality indicators that could be used in measuring the impact of pharmacists in providing care to patients in integrative care facilities, especially from the viewpoints of the stakeholders. As policy makers in health authorities are continuously under pressure to make decisions relevant to allocation of resources, improving quality of services, bridging gaps, attracting funds, improving accountability, and patient safety, exploring the viewpoints of stakeholders who could be healthcare providers (physicians, CAM practitioners, pharmacists, and nurses) or decision makers in managerial positions on quality indicators of pharmaceutical services becomes imperative.

This qualitative exploratory study was conducted to explore the views of different stakeholders who were physicians, CAM practitioners, pharmacists, nurses, and a risk/quality assurance manager on activities and services that could serve as quality indicators of pharmaceutical care in integrative healthcare facilities.

## 2. Methods

### 2.1. Study Context

This qualitative study was part of a larger project titled “Benchmarking Pharmaceutical Care in Integrative Healthcare Facilities.” The project aimed to develop a core list of consensual activities and services that could be used to measure and improve quality of pharmaceutical care in integrative healthcare facilities. The project was in three phases. In the first phase, the literature was reviewed with the aim of identifying, describing, and summarizing different activities and services that could be used to capture the impact of pharmacists in integrative healthcare facilities [24]. In the second phase, views of stakeholders on activities and services that could serve as quality indicators of pharmaceutical care in integrative healthcare facilities were explored. This manuscript reports the qualitative phases of the project. In the third phase, consensus-based activities and services that could be used as key performance indicators to capture and measure the impact of pharmacists in integrative healthcare facilities were developed.

### 2.2. Study Design

This qualitative exploratory study is being reported in compliance with the Consolidated Criteria for Reporting Qualitative Research (COREQ) checklist [25]. Details of adherence to the COREQ criteria are in Supplementary Table S1. In the present study, stakeholders were recruited and interviewed using in-depth semistructured interviews under naturalistic conditions to expose their viewpoints on what activities and services can be used as quality indicators of pharmaceutical care in integrative healthcare.

### 2.3. Sampling and Determination of the Sample Size

A judgmental sampling technique was used to invite and recruit interviewees for this study [26–28]. Potential interviewees were identified through personal contacts in the domain. Potential interviewees were approached, invited, and recruited based on their prior knowledge and experience in integrative medicine, pharmaceutical care, provision of pharmaceutical services, and measuring quality indicators. Healthcare providers (CAM practitioners, pharmacists, physicians, and nurses) with knowledge of the integrative medicine paradigm of healthcare delivery, having more than 5 years of practical experience, willing to give an informed consent, and willing to participate in a recorded interview were included in this study. Providers with less than 5 years of practical experience, unwilling to give an informed consent or unwilling to participate in a recorded interview, were excluded.

In this study, the thematic saturation point was used to estimate the sample size that was needed. The recruitment endpoint was decided a priori and was the point when sufficient conceptual themes, subthemes, and patterns were collected [28–30].

Based on previous studies with similar resource limitations and required quality standards, approximately 10 hours of interview time were needed [19, 27, 28, 30–38]. With a median interview time of approximately 40 minutes, a minimum of 15 interviews were required.

### 2.4. Recruitment

Potential participants were initially invited through emails and telephone calls. Initial invitations were sent to 27 potential participants. Invitations detailed the study design and the objectives. Nonrespondents received 3 reminders, each was 1 week apart.

### 2.5. Data Collection

The semistructured in-depth interviews were conducted by the main investigator (male, PhD, associate professor) who was employed by a major teaching university in Palestine who had prior knowledge and practical experience in moderating interviews and conducting qualitative research. Interviews were conducted in one-on-one sessions. Before the interviews, each interviewee was reminded that the interview was conducted for scientific research and the far goal of the interviews was to identify activities and services that might potentially be used as quality indicators. Additionally, each interviewee was assured that the investigators had no personal interests in the final outcomes of the study. The interviewees provided their sociodemographic and practice characteristics. After that, the interviews commenced with open-ended questions to collect all activities and services that the interviewees thought could be potentially used to capture the impact of pharmacists while caring for patients admitted to or visiting healthcare facilities that employ integrative medicine. The interviews were guided by an interview schedule which was developed and pilot-tested for this study. The interview schedule is presented in Supplementary Table S2. On occasions, the interviewer brought activities and services mentioned by other interviewees. These prompts were shown to give the interviewees opportunities to “hitch-hike” or catch-up with other participants and could have stimulated identification of more potential activities and services. The interviewees were audio-recorded and then transcribed verbatim. Field notes were also made by the investigator during the interviews. Transcripts were returned to the interviewees for review and comments.

### 2.6. Data Analysis

The qualitative analysis was based on the interpretive description methodology [39]. This methodology is useful in facilitating recognition of themes and patterns. The methodology was proven useful for interpreting descriptions across cases with complex experiential queries in healthcare [40]. In this study, the Qualitative Analysis Guide of Leuven was applied in recognizing themes, subthemes, and patterns [41]. Contents of the interviews were analyzed after listening to each interview at least three times. Potential activities and services were interpreted from the contents of the interviews [36]. Comprehension of the contents of the interviews and meaning of the data collected facilitated theorizing data points [42]. The data points collected were contextualized based on the theorized relationships between them. The data were coded using RQDA which is an open-source R package for Qualitative Data Analysis [43].

Although the interviewees spoke in Arabic language during the interviews, they were more comfortable stating key terms relevant to activities and services in English. This was because the language of instruction and teaching in professional and technical programs such as medical, pharmacy, and nursing schools was English. All interviewees were fluent in English. Interviews were conducted only once and were not repeated. Analysis was repeated over and over until all potential activities and services were obtained. Interpreted activities and services were quoted in both Arabic and English. Forward and back translations were performed. Activities and services quoted as original and translated were returned for each interviewee for feedback and correction. The interviewees were provided with another opportunity to add more potential activities and services if they wished to do so. The interviewees were identified and recruited from different healthcare facilities, private practice, regulatory bodies, and academic institutions in Palestine.

## 3. Results

### 3.1. Interviews and Response Rate

Of the 27 potential interviewees initially invited, 25 (92.6%) were willing to take part in the study and 2 (7.4%) declined for undisclosed reasons. However, interviews were conducted with 22 (81.5%) participants at the end of the study. Interviews were not scheduled with 5 (18.5%) potential participants due to unavailability or lack of time.

### 3.2. Sociodemographic and Practice Characteristics of the Interviewees

The interviewees were 9 CAM practitioners, 8 pharmacists, 2 physicians, 2 nurses, and 1 risk/quality assurance manager. Interviews were conducted with participants from all specialties intended to be represented in the study. Details of the sociodemographic and practice characteristics of the interviewees are shown in Table 1. The interview median duration was 41 with an IQR of 22 min.

### 3.3. Quality Indicators: Themes, Subthemes, and Patterns

There were many activities and services that pharmacists could provide in integrative medicine. The interviewees thought that many of these activities and services could serve as quality indicators of pharmaceutical care in integrative healthcare facilities. Following the thematic analysis adopted to achieve the objectives of this study, six major themes emerged from the data collected from the interviews. The themes emerged from the data were (1) provision of collaborative, direct, and comprehensive patient care services; (2) common services and activities at the time of admission, during stay, at transition between wards/services/hospitals, and at discharge to home or community care; (3) screening for, identifying, and resolving problems; (4) collaboration with other healthcare providers; (5) professional development; and (6) performance and efficiency. Details of these themes are shown in Table 2. The themes, subthemes, and patterns collected in this study represented personal accounts of the different stakeholders on quality indicators of pharmaceutical care in integrative healthcare.

#### 3.3.1. Provision of Collaborative, Direct, and Comprehensive Patient Care Services

In this study, the interviewees stated that pharmacists can provide collaborative, direct, and comprehensive patient care services to those admitted to integrative healthcare facilities. These services were related to taking the best possible therapy history, developing care plans, and assessing response to therapy.

The interviewees stated that pharmacists in integrative healthcare facilities are well placed to take the best possible therapy history from the patients admitted to these facilities. This service is often provided at the time of admission to know what medications and CAM modalities the patient was and/or is currently using at the time of admission. Healthcare providers might need such details to inform optimal healthcare services to the patients as some of these medications and CAM modalities might be continued, modified, or stopped during the duration of patient's stay in the facility. One interviewee shared the following:

“Pharmacists are experts on medications. They also know about CAM. I think they are the best to take the best history from the patient on what medications and forms of CAM they have or might be using. This can help inform better decisions…” A pharmacist.

The interviewees also reported that pharmacists should be actively involved in developing, implementing, and monitoring care plans for patients admitted to integrative healthcare facilities. Pharmacists might make recommendations relevant to the medications and/or CAM modalities, the optimal doses, frequency, and duration of use. One of the interviewees shared the following:

“…pharmacists have always recommended medications to patients. In some countries, pharmacists were permitted even to prescribe medications. I am not talking about those sold over-the-counter here, you know what I mean?……in clinical practice, pharmacists should share their opinion on what therapy to be used, how much of it, and for how long.” A pharmacist.

After implementation, pharmacists might take a more active role in following up with patients and monitoring their care plans. Here, pharmacists might make recommendations to change medications and/or CAM modalities, doses, frequencies, and durations. Pharmacists might order tests and review their results before they can make better informed decisions. The same stakeholder continued the following:

“…the role of pharmacist should not stop here. The pharmacist should follow up with the treatment plan and make recommendations. Lab tests and results are often available and handy to inform decisions. They should be used.” A pharmacist.

The interviewees also reported that response to therapy should be assessed and documented by the pharmacist. One interviewee shared the following:

“…documentation is also important. The pharmacist should assess the treatment plan if it is working or not, you know what I mean?” A nurse.

Based on their assessments, pharmacists should also make recommendations to achieve the desired therapeutic outcomes. One interviewee shared the following:

“…after assessment, if it turned out that the dose was not optimal, the dose might be increased or decreased to help achieve the desired outcomes. This is important!” A CAM practitioner.

#### 3.3.2. Common Services and Activities at Admission, during Stay, at Transition of Care, and Discharge

Pharmacists can provide many services and activities at the different phases of patient care. In this study, the interviewees mentioned services and activities that could be common at the time of admission, during stay, at transition between wards/services/hospitals, and at discharge of the patient to home or community care.

At all stages of patient care, pharmacists should perform periodic review of the therapeutic options used, either medications or CAM modalities. One interviewee shared the following:

“…medication review is one of the most important services provided by the pharmacist. The pharmacist's expertise are indispensable when it comes to medication reviews”. A physician.

Pharmacists should also perform reconciliation services to ensure that patients know their therapies and are using them according to the care plan. One interviewee shared the following:

“…medication reconciliation should be performed at time of admission, when the patient is moved from one facility to another, and at the time when the patient is to be discharged. We need to minimize medication errors and discrepancies.” A pharmacist.

The interviewees also stated that pharmacists are in key position to counsel patients and educate about their therapies. One interviewee shared the following:

“…educating patients should be ensured. On many occasions, patients need to know more about their medications. Patients always have questions and complaints. I think no one is in a better position to educate patients and answer their therapy relevant questions than the pharmacist!” A pharmacist.

### 3.4. Screening for, Identifying, and Resolving Problems

The interviewees also stated that the pharmacists should be actively involved in screening for, identifying, and resolving problems related to therapies. These problems could be related to interactions, inappropriate use, adverse reactions, and problematic orders.

Some medications can interact with other medications, foods, and CAM. Such interactions might result in detrimental consequences to the health of the patient. The interviewees stated that pharmacists should screen for, identify, and resolve these interactions. One interviewee shared the following:

“…pharmacists are experts on pharmacotherapy. They know more than other healthcare professionals what medications do interact with each other and what medications do not. Interactions could occur between different medications themselves, foods, or CAM. I think pharmacists should be able to resolve these interactions.” A physician.

The interviewees stated that pharmacists should screen for inappropriate use of therapeutic options. For example, the dose could be too high, too low, or inappropriate for the patient's renal or hepatic function. One interviewee shared the following:

“…in clinical practice, the patient could be underdosed or overdosed. The pharmacist should also assess if the dose is suitable for the patient's renal function. We need to make sure that the medication is being eliminated and does not accumulate to toxic levels in the body of the patient” A CAM practitioner.

The interviewees indicated that more than one medication or CAM modality could be prescribed for the same indication. This could be unnecessary and might predispose the patient to adverse reactions. One interviewee shared the following:

“…for the same condition, the prescriber could opt for many options. Therefore duplication is very common. I think the pharmacist should be vigilant and apply the necessary measures.” A risk/quality assurance manager.

Therapies used could be ineffective sometimes. As other alternative therapeutic options are available, they could also be recommended. One interviewee shared the following:

“…if the desired outcomes are not being met, it could be wise to assess the effectiveness of the therapy used. Why ineffective therapies should be continued?” A CAM practitioner.

The use of some medications and CAM could be contraindicated for some patients. Pharmacists could be indispensable in making appropriate recommendations to avoid using contraindicated therapies. One interviewee shared the following:

“…therapies should be assessed for suitability of use in certain patients. In case the medication was contraindicated, the pharmacist should make recommendation for a better option” A CAM practitioner.

Many patients exhibit allergies to medications and different CAM modalities. The interviewees stated that pharmacists are better placed to recommend alternatives in case the patient exhibited allergy to therapy. One interviewee shared the following:

“…in case of allergy to medications, the pharmacist should be prepared to be asked for an alternative. There are many alternatives that pharmacists could suggest.” A pharmacist.

Although adverse reactions are often seen with some therapeutic options, however, sometimes they can be avoided or minimized. The interviewees stated that pharmacists should identify adverse reactions and revolve them whenever possible. One interviewee shared the following:

“…as the therapy has many benefits to the patients, they can also be associated with unwanted effect. Although patients are encouraged to cope with the side effects of the therapy, however, it is not always wise to ignore them, especially when they become severe or when better alternatives are handy.” A CAM practitioner.

Pharmacists also are supposed to resolve problematic orders such as ambiguous orders, illegibly written orders, and misspelled medication names. Such problematic orders can lead to medication errors which can result in patient harm. This can be particularly true in healthcare systems where orders are paper-based. One interviewee shared the following:

“…I know that misspelling medication names and illegibly written medication orders can lead to errors. Pharmacists should pay close attention to orders and try to clarify those with ambiguities.” A CAM practitioner.

Many orders could be missing important information such as name of the drug, dose, frequency, route of administration, duration of therapy, or recommendations to take therapy in relation to meal. Pharmacists should screen orders for completeness and resolve problems that could be associated with missing orders. One interviewee shared the following:

“…missing orders are common. I have received many orders in which the prescriber did not indicate the dose. I was confused because the medication was available in multiple strengths.” A pharmacist.

#### 3.4.1. Collaboration with Other Healthcare Providers

Multihealthcare provider paradigms are predominant in modern healthcare delivery. In daily practice, pharmacists are constantly required to collaborate with other healthcare providers which might require attending interprofessional meetings, taking part in professional discussions, making suggestions, and answering formal inquiries. One interviewee shared the following:

“…the presence of pharmacists in interprofessional meetings and discussions should be strong. Pharmacists should lead discussions and provide suggestions. They are main decision makers across the continuum of the healthcare provision process.” A pharmacist.

It is common for pharmacists to receive formal inquiries from other healthcare providers. Pharmacists should demonstrate their expertise as partners to other healthcare professionals such as physicians, nurses, and CAM practitioners. One interviewee shared the following:

“…pharmacists should demonstrate their expertise as partners to other healthcare professionals like physicians, nurses, and CAM practitioners. Those professionals make formal inquiries to pharmacists. These inquiries need to be answered in the best way.” A risk/quality assurance manager.

#### 3.4.2. Professional Development

The interviewees stated that pharmacy is an ever evolving profession. Pharmacists should keep an eye on professional development. One interviewee shared the following:

“…there is always something new that you need to know. Pharmacy is a continually evolving profession. Roles and responsibilities of pharmacists are expanding. I think professional development through continuing education should be given a high priority.” A pharmacist.

The interviewees pointed to the newly evolving roles and responsibilities of pharmacists. The interviewees also referred to the willingness of the pharmacists to be actively involved in providing collaborative and direct patient care. As pharmacists might need to perform new activities and provide new services, continuing education might be necessary.

#### 3.4.3. Performance and Efficiency

Provision of quality care implies freedom of errors which might be associated with satisfaction of the consumers. One interviewee shared the following:

“…there is no system that is absolutely error-free. Needless to say that committing more errors can be detrimental to the professional reputation and proficiency.” A CAM practitioner.

Another interviewee added the following:

“…listening to the opinions of the consumers is very important. Today, decision makers take consumer complaints seriously. In case of any complaint, corrective actions should be initiated immediately. I believe the same complaints should not be received again.” A CAM practitioner.

The interviewees pointed out that provision of services should be as error-free as possible. Although no system is absolutely error-free, however, errors, especially medication errors should be minimized. Again, in the opinion of the interviewees, patient satisfaction with activities and services provided by pharmacists was also important.

## 4. Discussion

### 4.1. Summary of the Main Findings of the Study

This qualitative study sought to explore views of different stakeholders on activities and services that could be used as quality indicators of pharmaceutical care in integrative healthcare facilities. CAM practitioners, pharmacists, physicians, nurses, and a risk/quality assurance manager were interviewed. Findings of this study showed that many activities and services that could be used as quality indicators of pharmaceutical care in integrative healthcare facilities were suggested by the stakeholders. Activities and services suggested by the interviewees were categorized into themes, subthemes, and patterns. The major themes emerged from this study were related to providing care, services at admission, stay, transition, and discharge, resolving therapy-related problems, collaboration with others, professional development, and efficiency in service provision. This is the first investigation to explore views of stakeholders on quality indicators of pharmaceutical care in integrative healthcare facilities.

### 4.2. Appraisal of the Methods Used in This Study

In this qualitative exploratory study, semistructured in-depth interviews were used. Interviews were previously used as useful means in exposing views and perspectives of those with extensive experience in the field [19, 26, 27, 32–36, 44, 45]. Interviews conducted in this study were guided by an interview schedule. The interview schedule was developed and piloted. Piloting the interview schedule could have imparted strength to the methods used in this study. The interviewees were recruited using a judgmental sampling technique. This sampling approach ensured reaching out to the interviewees, diversifying the recruited sample, and the data collected from the interviews [19, 26, 27, 32–35]. Interviews were conducted in one-on-one sessions and the interviewer possessed previous experience in conducting qualitative studies. This might have avoided domination of one interviewee over the rest of the participants as could happen in group interviews. The data collected in this study were analyzed using the interpretive description methodology [39]. Previous studies have shown that this method is especially powerful in recognizing themes while interpreting qualitative data from cases with complex and experiential questions in healthcare [30, 40].

### 4.3. Activities and Services Performed by the Pharmacist

In this study, the interviewees suggested using provision of collaborative, direct, and comprehensive patient care services by the pharmacists as quality indicators in integrative care. Findings of this study were consistent with those reported in previous studies in which the roles of pharmacists as healthcare providers were recognized [19–22]. In today's healthcare systems, pharmacists take medication histories, develop, implement, and monitor care plans, and assess response to therapy [19]. The interviewees in this study also mentioned some activities and services that the pharmacist could perform at different stages across the continuum of healthcare (at admission, during stay, at transition between wards/services/hospitals, and at discharge to home or community care). Many previous studies have demonstrated that pharmacist-led medication reconciliation, review, and counselling can improve clinical outcomes at hospital transitions and reduce drug-related readmissions [46–49]. Screening for, identifying, and resolving problems were also mentioned by the interviewees as activities and services that could be used as quality indicators of pharmaceutical care in integrative healthcare facilities. These findings were consistent with those reported previously in which activities and services such as resolving problems related to antiepileptic drugs were considered quality indicators that could be used to measure the impact of pharmacists in providing healthcare for patients with epilepsy [19]. Inclusion of pharmacists in the healthcare team has proven effective in reducing medication-related problems such as interactions, inappropriate use, adverse medication reactions, and problematic orders [49–54]. Similarly, pharmacists in integrative healthcare facilities are supposed to screen medication orders for completeness, interactions, inappropriate use, and risk of adverse medication reactions. The interviewees also mentioned that pharmacists are supposed to attend interprofessional meetings and actively participate in professional discussions. The competencies of pharmacists as experts in pharmacotherapy are well recognized among the multiprovider healthcare delivery team. Recent studies have shown that the vast majority of pharmacists' recommendations and suggestions are accepted by the physicians and other healthcare providers [55–57]. In this study, the interviewees also mentioned that professional development through continuing education and training were also important to take into consideration as quality indicators. Findings of this study were in contrast with those reported in a previous study in which a panel of experts did not consider attending continuing education a key performance indicator of pharmaceutical care of patients with epilepsy [19]. However, pharmacy is a dynamic profession. Therefore, the roles and responsibilities of pharmacists are continuously expanding. Continuing education and training for pharmacists have been backed up by many professional groups. Universities and training institutions are adapting to the needs of busy learners and offering different ways to keep pharmacists updated with latest information [58–61]. Performance and efficiency in provision of services have emerged as important indicators of quality in many service sectors including healthcare. In this study, the interviewees mentioned that complaints about activities and services and errors committed by pharmacists could be used as quality indicators. These findings were consistent with those reported by Lloyd et al. in which dissatisfaction of the consumers could be used to benchmark services [62]. Both complaints and errors committed by the pharmacists could be used in improving activities and services provided by the pharmacists in integrative healthcare facilities [34, 35, 44]. However, findings of this study were contradictory to those previously reported in a previous study in which consensus was not achieved to consider the number of errors committed by pharmacists as a performance indicator of pharmaceutical care of patients with epilepsy [19].

It is noteworthy mentioning that not all activities and services can be used in measuring and improving the quality of pharmaceutical care in healthcare facilities including those employing integrative care. Therefore, researchers and quality assurers often have to prioritize activities and services that could be used to benchmark pharmaceutical care in healthcare facilities. Activities and services provided by the interviewees in this study should have enriched those collected from the literature in the first phase of the larger project [24]. Another phase is still needed to develop a core list of consensus-based activities and services that could be used as key performance indicators to capture and measure the impact of pharmacists in integrative healthcare facilities.

### 4.4. Strengths and Limitations of the Study

Findings of this study might be interpreted after taking into consideration the following strengths and limitations. First, this study was a qualitative study in which stakeholders were interviewed. Adding a quantitative dimension could have further strengthened our findings. Second, all interviewees were from Palestine. This might further limit the generalizability of the findings. Third, the number of interviewees was relatively limited. The number of interviewees needed for this study was determined by the thematic saturation point. It is noteworthy mentioning that the interviewees were CAM practitioners, pharmacists, physicians, nurses, and a risk/quality assurance manager. The interviewees were of both genders, had different academic backgrounds, were employed in different sectors, and had long experience in the field. This might have added validity to the findings obtained in this study. Fourth, patients attending integrative healthcare facilities were not interviewed in this study. Exposing perspectives of the patients as utilizers of the services could be important in informing decisions to benchmark quality. Finally, data analysis was based on the interpretive description methodology. This method is associated with many challenges such as limited resources, limitation of using a less-known method, and lesser certainty concerning the degree of interpretation sought [63].

## 5. Conclusions

This explorative qualitative investigation reported perspectives of different stakeholders on activities and services that could be used as quality indicators of pharmaceutical care in integrative healthcare facilities. Quality indicators are invaluable for informing decisions relevant to justifying allocation of scarce resources, securing funds, and demonstrating value in activities and services within integrative healthcare facilities. Further studies are still needed to develop a set of measurable indicators to measure the impact of pharmaceutical care in integrative healthcare facilities.

## Figures and Tables

**Table 1:** Sociodemographic and practice characteristics of the interviewees (*n* = 22).

Characteristic	*n*	%
Gender		
Male	14	63.6
Female	8	36.4

Age (years)		
<40	16	72.7
≥40	6	27.3

Profession		
CAM practitioner	9	40.9
Pharmacist	8	36.4
Physician	2	9.1
Nurse	2	9.1
Risk/quality assurance manager	1	4.5

Academic degree		
BSc	8	36.4
MSc	6	27.3
MD	2	9.1
PhD	6	27.3

Employer		
Hospital	11	50.0
Private practice	3	13.6
Pharmacy	2	9.1
Educational/training organization	5	22.7
Regulatory body/ministry	1	4.5

Length of practical experience in the domain (years)		
5–10	9	40.9
≥10	13	59.1

BSc, bachelor of science; CAM, complementary and alternative medicine; MD, doctor of medicine; MSc, master of science; PhD, doctor of philosophy.

**Table 2:** Thematic analysis of interviews (*n* = 22).

Themes	Subthemes	Patterns	Example of a representative quotation
Provision of collaborative, direct, and comprehensive patient care services	Best possible therapy history	Taking history	“…pharmacists are supposed to see the patients at the time of admission and talk with them about the medications and other therapies they have used. This might help us decide what we can try later and it might give us an idea if that is going to work or not”
	Care plans	Developing care plans	“…treatment plans should not be decided by physicians alone. Pharmacists should assume more active role and share their recommendations with regard to the medication, dose, and for how long it should be used”
		Implementing and monitoring care plans	“…the role of pharmacists should not stop here. The pharmacists should follow up with the treatment plan and make recommendations. Lab tests and results are often available and handy to take decisions. They should be used”
	Response to therapy	Documenting assessments of response to therapeutic plan	“…if it was not written it was not done! I think pharmacists should assess and document what they have assessed. This can facilitate decision making”
		Titration of doses to produce desirable therapeutic effect	“…in case we are not using the right therapy or the right doses, this has to be changed accordingly until we arrive at the desired outcomes whenever possible”

Common services and activities at the time of admission, during stay, at transition between wards/services/hospitals, and at discharge to home or community care	Review and reconciliation	Review	“…pharmacists should perform periodic medication reviews. We need to make sure that patients are making the best of their medications”
		Reconciliation	“…pharmacists are good at performing medication reconciliation services. We need to know that the patients know their medications and are using them as planned”
	Counselling and educating patients		“Pharmacists should counsel the patients on their medications. Counselling has proven effective in increasing adherence and coping with side effects”
Screening for, identifying, and resolving problems	Interactions	Medications	“…pharmacists should ensure that the medications used do not interact with each other”
		CAM	“…like medications, CAM oftentimes might interact with other CAM or medications”
		Food	“…pharmacists often inform patients what foods to take or avoid when taking medications. This is part of their patient instructions”
	Inappropriate use	Inappropriate dose	“…the patient could be underdosed or overdosed. The pharmacist should also assess if the dose is suitable for the patient's renal function. We need to make sure that the medication is being eliminated and does not accumulate to toxic levels in the body of the patient”
		Duplication	“…for the same condition, the prescriber could opt for many options. Therefore, duplication is very common. I think the pharmacist should be vigilant and apply the necessary measures”
		Ineffectiveness	“…therapeutic options are not always effective. Pharmacists should monitor if the patient is responding to the therapy used or not”
		Contraindications	“…therapies should be assessed for suitability of use in certain patients. In case the medication was contraindicated, the pharmacist should make recommendation for a better option”
		Allergies	“…in case of allergy to medications, the pharmacist should be prepared to be asked for an alternative. There are many alternatives that pharmacists could suggest”
	Adverse reactions		“…as the therapy has many benefits to the patients, they can also be associated with unwanted effect. Although patients are encouraged to cope with the side effects of the therapy, however, it is not always wise to ignore them, especially when they become severe or when better alternatives are handy”
	Problematic orders	Ambiguous order	“…therapeutic orders could be ambiguous and unclear. Pharmacists should talk to the prescribers and clarify them before any action can be taken”
		Illegibly written order	“…when orders are handwritten, writing can be very important. Illegibly written orders can be a source of errors”
		Misspelled order	“…when a prescriber misspells the medication name, this can be confused with another medication. There are many sound-alike and look-alike medications”
		Missing order	“…missing orders are common. I have received many orders in which the prescriber did not indicate the dose. I was confused because the medication was available in multiple strengths”
Collaboration with other healthcare providers	Meetings	Attending interprofessional meetings	“…meetings can be numerous and time consuming. However, pharmacists should spare some time and attend them”
		Taking part in professional discussions	“…decision making is a shared responsibility now. No professional group should dominate the discussion. We are all partners now”
		Making suggestions to other healthcare providers	“…pharmacists suggestions are more encouraged and attended from other healthcare professionals now”
	Inquiries	Answering formal inquiries	“…it takes a lot of efforts to formally answering questions and inquiries of other healthcare professionals in the team”

Professional development		Attending continuing educational/training	“Pharmacists should attend periodic continuing education and training. In many countries, this has been set as a requirement for relicensure”
		Providing continuing educational/training	“…pharmacists are good educators. They always educate patients. They can also educate each other junior pharmacists as well as other healthcare professionals”

Performance and efficiency		Errors committed	“…there is no system that is absolutely error-free. Needless to say that committing more errors can be detrimental to the professional reputation and proficiency”
		Complaints received	“…listening to the opinions of the consumers is very important. Today, decision makers take consumer complaints seriously. In case of any complaint, corrective actions should be initiated immediately. I believe the same complaints should not be received again”

CAM, complementary and alternative medicine.

## Data Availability

Data supporting the results reported in a published article can be found in the Results section and as Supplementary Materials with this manuscript.

## Supplementary Materials

Supplementary Materials Supplementary Table S1: adherence to Consolidated Criteria for Reporting Qualitative Research (COREQ) checklist. Supplementary Table S2: the interview schedule.
