# Supplementary material for: Quality Indicators of Pharmaceutical Care in Palestinian Integrative Healthcare Facilities: Findings of a Qualitative Study among Stakeholders
Source: Evid Based Complement Alternat Med. 2020 May 10;2020:4520769. doi: 10.1155/2020/4520769 (PMC7238345; doi:10.1155/2020/4520769)
Supplement: Supplementary Materials — Supplementary Table S1: adherence to Consolidated Criteria for Reporting Qualitative Research (COREQ) checklist. Supplementary Table S2: the interview schedule. [file 4520769.f1.docx]

**Supplementary Table S1:** Adherence to COnsolidated criteria for REporting Qualitative research (COREQ) Checklist [[1](#_ENREF_1)]

| **#** | **Topic** | **Guide Questions/Description** | **Page/Line # in the manuscript** |
| --- | --- | --- | --- |
|  | **Domain 1: Research team and reﬂexivity** |  |  |
|  | *Personal characteristics* |  |  |
| 1 | Interviewer/facilitator | Which author/s conducted the interview or focus group? | Page 6 Line 2-4 |
| 2 | Credentials | What were the researcher’s credentials? E.g. PhD, MD | Page 6 Line 2-4 |
| 3 | Occupation | What was their occupation at the time of the study? | Page 6 Line 2-4 |
| 4 | Gender | Was the researcher male or female? | Page 6 Line 2-4 |
| 5 | Experience and training | What experience or training did the researcher have? | Page 6 Line 2-4 |
|  | *Relationship with participants* |  |  |
| 6 | Relationship established | Was a relationship established prior to study commencement? | Page 6 Line 4-8 |
| 7 | Participant knowledge of the interviewer | What did the participants know about the researcher? e.g. personal goals, reasons for doing the research | Page 6 Line 4-8 |
| 8 | Interviewer characteristics | What characteristics were reported about the interviewer/facilitator? e.g. Bias, assumptions, reasons and interests in the research topic | Page 6 Line 4-8 |
|  | **Domain 2: Study design** |  |  |
|  | *Theoretical framework* |  |  |
| 9 | Methodological orientation and Theory | What methodological orientation was stated to underpin the study? e.g. grounded theory, discourse analysis, ethnography, phenomenology, content analysis | Page 6 Line 20-28 |
|  | *Participant selection* |  |  |
| 10 | Sampling | How were participants selected? e.g. purposive, convenience, consecutive, snowball | Page 5 Line 20-31 |
| 11 | Method of approach | How were participants approached? e.g. face-to-face, telephone, mail, email | Page 5 Line 36-38 |
| 12 | Sample size | How many participants were in the study? | Page 5 Line 36-38 and Page 8 Line 3-6 and Table 1. |
| 13 | Non-participation | How many people refused to participate or dropped out? Reasons? | Page 8 Line 3-6 |
|  | *Setting* |  |  |
| 14 | Setting of data collection | Where was the data collected? e.g. home, clinic, workplace | Page 6 Lines 2-18 |
| 15 | Presence of non-participants | Was anyone else present besides the participants and researchers? | N/A |
| 16 | Description of sample | What are the important characteristics of the sample? e.g. demographic data, date | Page 8 Line 8-12 and Table 1 |
|  | *Data collection* | | |
| 17 | Interview guide | Were questions, prompts, guides provided by the authors? Was it pilot tested? | Page 6 Lines 12-13 and Supplementary Table S2 |
| 18 | Repeat interviews | Were repeat interviews carried out? If yes, how many? | Page 6 Line 31-32 |
| 19 | Audio/visual recording | Did the research use audio or visual recording to collect the data? | Page 6 Line 16-18 |
| 20 | Field notes | Were ﬁeld notes made during and/or after the interview or focus group? | Page 6 Line 16-18 |
| 21 | Duration | What was the duration of the interviews or focus group? | Page 8 Line 8-11 |
| 22 | Data saturation | Was data saturation discussed? | Page 5 Lines 29-31 |
| 23 | Transcripts returned | Were transcripts returned to participants for comment and/or correction? | Page 6 Line 34-38 |
|  | **Domain 3: analysis and ﬁndings** |  |  |
|  | *Data analysis* | | |
| 24 | Number of data coders | How many data coders coded the data? | Page 6 Line 29-38 |
| 25 | Description of the coding tree | Did authors provide a description of the coding tree? | N/A |
| 26 | Derivation of themes | Were themes identiﬁed in advance or derived from the data? | Page 6 Line 20-28 |
| 27 | Software | What software, if applicable, was used to manage the data? | Page 6 Line 26-28 |
| 28 | Participant checking | Did participants provide feedback on the ﬁndings? | Page 6 Line 34-38 |
|  | *Reporting* |  |  |
| 29 | Quotations presented | Were participant quotations presented to illustrate the themes/ﬁndings? Was each quotation identiﬁed? e.g. participant number | Table 2 and Page 12-15. |
| 30 | Data and ﬁndings consistent | Was there consistency between the data presented and the ﬁndings? | N/A |
| 31 | Clarity of major themes | Were major themes clearly presented in the ﬁndings? | Table 2 and Page 12-15. |
| 32 | Clarity of minor themes | Is there a description of diverse cases or discussion of minor themes? | Table 2 and Page 12-15. |

**Reference**

1. A. Tong, P. Sainsbury, and J. Craig, "Consolidated criteria for reporting qualitative research (COREQ): a 32-item checklist for interviews and focus groups," *Int J Qual Health Care*, vol. 19, no. 6, pp. 349-357, 2007.

**Supplementary Table S2:** Interview schedule

| **#** | **Question*** |
| --- | --- |
| 1 | Could you please tell me about the importance of pharmacists as healthcare providers in integrative healthcare facilities? |
| 2 | Could you please tell me about the care activities and services that pharmacists could provide in integrative healthcare facilities? |
|  | - At admission? |
|  | - During stay? |
|  | - At transition of care? |
|  | - At discharge? |
| 3 | Could you please elaborate on the problems that pharmacists could solve while caring for patients in integrative healthcare facilities? |
| 4 | What collaborations pharmacists are supposed to establish with other healthcare providers in the team? |
| 5 | What pharmacists can do improve their performance in providing optimal care to patients in integrative healthcare facilities? |
| 6 | What activities and services can be used as quality indicators of pharmaceutical care in integrative healthcare facilities? |

*Prompts like how? Why? Could you please explain? Could you please elaborate? were occasionally used whenever the interviewer felt needed.
